# Remote Contouring and Virtual Review during the COVID-19 Pandemic (RECOVR-COVID19): Results of a Quality Improvement Initiative for Virtual Resident Training in Radiation Oncology

**DOI:** 10.3390/curroncol28040259

**Published:** 2021-08-05

**Authors:** Andrew J. Arifin, Rohann J. M. Correa, Christopher D. Goodman, Joanna Laba, Robert E. Dinniwell, David A. Palma, Timothy K. Nguyen

**Affiliations:** Division of Radiation Oncology, London Regional Cancer Program, London, ON N6A 5W9, Canada; Andrew.Arifin@lhsc.on.ca (A.J.A.); Rohann.Correa@lhsc.on.ca (R.J.M.C.); Chris.Goodman@lhsc.on.ca (C.D.G.); Joanna.Laba@lhsc.on.ca (J.L.); Robert.Dinniwell@lhsc.on.ca (R.E.D.); David.Palma@lhsc.on.ca (D.A.P.)

**Keywords:** medical education, virtual education, contouring, feedback, radiation oncology

## Abstract

The need to minimize in-person interactions during the COVID-19 pandemic has led to fewer clinical learning opportunities for trainees. With ongoing utilization of virtual platforms for resident education, efforts to maximize their value are essential. Herein we describe a resident-led quality improvement initiative to optimize remote contouring and virtual contour review. From April to June 2020, radiation oncology (RO) residents at our institution were assigned modified duties. We implemented a program to source and assign cases to residents for remote contouring and to promote and optimize virtual contour review. Resident-perceived educational value was prospectively collected and analyzed. All nine RO residents at our institution (PGY1–5) participated, and 97 cases were contoured during the evaluation period. Introduction of the Remote Contouring and Virtual Review (RECOVR) program coincided with a significant increase in mean cases contoured per week, from 5.5 to 17.3 (*p* = 0.015), and an increased proportion of cases receiving virtual review, from 14.8% to 58.6% (*p* < 0.001). Residents reported that the value of immediate feedback during virtual review was similar to that of in-person review (4.6 ± 0.1 vs. 4.5 ± 0.2, *p* = 0.803) and significantly higher than feedback received post hoc (e.g., email; 3.6 ± 0.2, *p* < 0.001). The implementation of a remote process for contour review led to significant increases in contouring, and virtual contour review was rated as highly as in-person interactions. Our findings provide a data-driven rationale and framework for integrating remote contouring and virtual review into competency-based medical education.

## 1. Introduction

Healthcare institutions worldwide have adapted operations amidst the ongoing COVID-19 pandemic, including modifications to the delivery of clinical services and medical education [1]. These changes have led to a decrease in clinical learning opportunities across multiple disciplines [2]. As a result of physical distancing measures, educational institutions have shifted to virtual learning platforms.

Literature on the use of virtual platforms in radiation oncology (RO) medical education during the pandemic is emerging. Experiences from two RO virtual medical student clerkships have been published [3,4]. The two programs incorporated virtual didactic sessions but differed in how experiential learning was conducted. One program incorporated medical students into telemedicine clinics, interacting with staff and patients virtually. The other gave students access to contouring software, though details regarding the contour review process were not provided. To our knowledge, there has only been one published initiative related to RO resident education during the pandemic [5], which described a pilot program for mock virtual oral examinations through video conferencing software.

Contouring is a core competency for RO trainees. Traditionally, trainee contours are reviewed in person, with staff editing contours while verbalizing their thought processes and rationale. This provides opportunities for direct observation, discussion, and immediate feedback. Seeing the edits in real time provides the trainee with insight into minor corrections that may not be verbalized. As in-person review is discouraged during the pandemic, these valuable learning opportunities are lost if programs do not adapt. Because it is a computer-based task, contouring is uniquely amenable to virtual platforms.

During the height of the first wave of the pandemic, residents were faced with a lack of educational opportunities, including contouring experiences. The goal of this project was to develop and assess a framework for virtual contour review to provide impactful resident teaching despite pandemic restrictions.

## 2. Materials and Methods

Prior to the pandemic, our training program followed apprenticeship-style rotations in which a resident was paired with one staff for 1 to 2 months and followed their schedule with respect to clinical and radiation planning work. Contour review was usually done in person with staff sitting down at a computer station one-on-one with the trainee, or feedback was given after the fact via email or over the phone. From April to June 2020, during the height of the first wave of COVID-19 cases, RO residents at our institution were assigned modified duties to ensure adequate coverage of clinical services and limit transmission risk. Formal educational activities were suspended, including lectures, grand rounds, and practice oral examinations. Prior to the implementation of the Remote Contouring and Virtual Review (RECOVR) program, residents were encouraged to approach staff in an ad hoc manner to discuss contouring assignments without any expectation regarding case volume or format of review. Most staff edited contours independently and provided residents with feedback after the fact. Residents reported a dramatic decline in the volume of cases contoured during this period.

To address the decrease in educational opportunities in our program and to increase the number of cases contoured, we implemented the RECOVR program for efficient sourcing and assignment of cases to resident learners. We felt that the decrease in learner engagement was due to a lack of direction regarding case procurement and a perceived decrease in value of the contour review process. We theorized that setting expectations and a framework for review would help increase resident activity and engagement.

**Figure 1:**
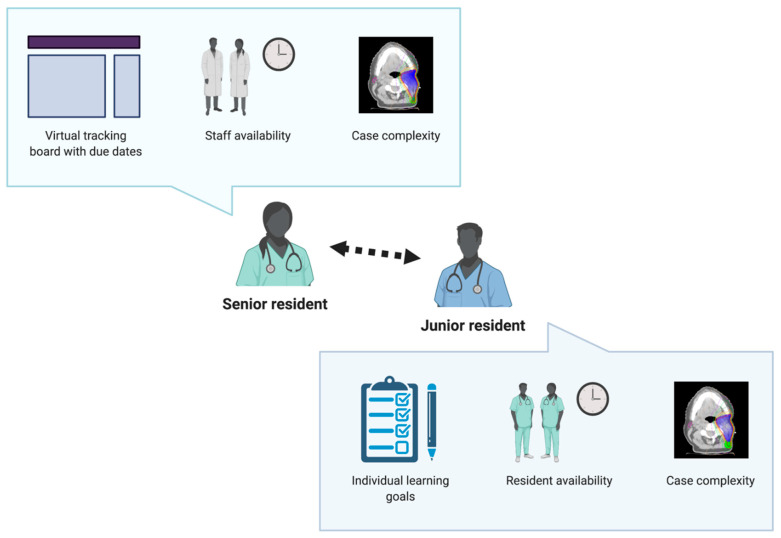
Mentorship model for case selection. Cases for resident learners were selected following discussion by senior and junior residents. Created with BioRender.com.

During the program, residents were expected to contour 2 or more cases per week. We developed a workflow using a virtual tracking board of all cases in the department awaiting planning (Figure 1). Team leaders (senior residents) used a mentorship model to assign cases to resident learners based on case priorities, staff and learner availability, and trainee level and learning goals. At the beginning of each week, residents would approach team leaders about their availabilities and if they had specific sites of interest. Team leaders reviewed the tracking board daily. Cases with short turnaround times were typically not assigned to residents, especially junior trainees. Team leaders would contact residents throughout the week with potential case assignments, aiming to ensure all residents met their weekly targets and sites of interest. Once agreed upon, team leaders contacted staff regarding the case assignment. Having team leaders as the liaison between staff and residents prevented multiple residents from attempting to contour the same cases.

**Figure 2:**
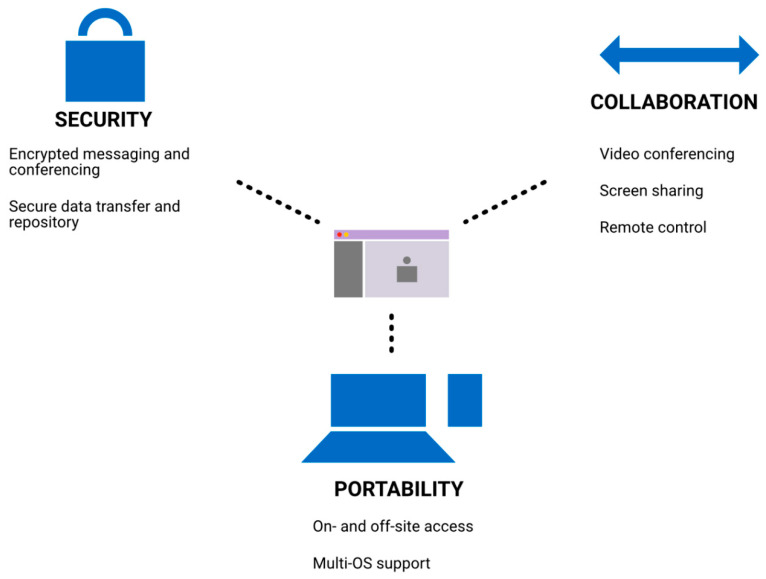
Virtual review software features. We identified three domains that allowed for the success of virtual contouring review, including security, collaboration, and portability.

We used the Microsoft Teams platform for virtual contour review (Figure 2). Security features and privacy compliance allowed for the exchange of patient-level information, while its multi-system support allowed for use on- and off-site on multiple devices. Screen sharing and video call features were used for the review of contours. The ability to give control to another user during screen sharing allowed both the staff and resident to edit contours at the same time.

After each contour review, residents were instructed to complete a case log. Data were prospectively captured to inform further quality improvement efforts and to optimize the RECOVR program. These data included the format of review (e.g., virtual, in-person, etc.), learning pearls from the case, and qualitative feedback about the program. Perceived educational value of the case overall and the educational value of the review process were captured on a 5-point Likert scale. Residents were asked if they felt that the case provided educational value and that the review process provided educational value. A score of 1 corresponded to “Strongly Disagree”, 3 “Neutral”, and 5 “Strongly Agree”. Based on feedback from trainees, we amended the case log form with an embedded link to our institution’s assessment platform. Descriptive and comparative statistics (Student’s *t*-test, chi-square, Fisher’s exact test, and ANOVA with multiple comparisons) were used to analyze the data. Thematic analysis of learning pearls was performed using the free text responses submitted by residents.

## 3. Results

**Figure 3:**
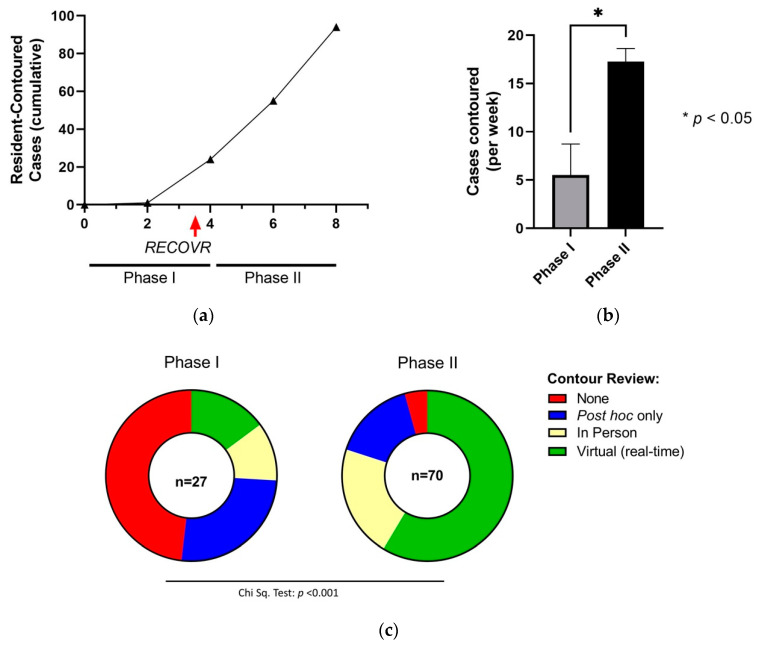
Outcomes as a result of the implementation of the RECOVR program. (**a**) Plot of cumulative resident-contoured cases as a function of weeks since April 1, 2020. (**b**) We observed a significant increase in the number of resident-contoured cases in Phase II compared to Phase I. (**c**) There was a significant difference in the proportion of formats of contour review between Phase I and II. * *p* < 0.05.

All nine RO residents (PGY1–5) at our institution participated in the RECOVR program. A total of 97 cases were contoured during the evaluation period. Before introducing the program (Phase I; 6 April 2020 to 1 May 2020), we observed a paucity of resident-contoured cases with only an average of 5.5 ± 3.3 (mean ± standard error) cases contoured per week by all residents combined or 0.7 ± 0.4 cases per week per resident, and only 14.8% (4/27) of cases receiving virtual review (i.e., real-time feedback). After RECOVR implementation (Phase II; 4 May 2020 to 4 June 2020), we documented an increase in case volume over time (Figure 3a) with 17.3 ± 1.4 cases contoured per week by all residents combined (*p* = 0.015, unpaired Student’s *t*-test, Figure 3b) or 1.9 ± 0.2 cases per week per resident. This was not associated with an increase in the number of cases contoured overall in the department, with 331, 377, and 381 cases planned in April, May, and June 2020, respectively. Moreover, a significant increase in the use of virtual contour review was observed (14.8% vs. 58.6%, *p* < 0.001, chi-squared test, Figure 3c). The proportion of in-person contouring review did not significantly change from Phase I to II (*p* = 0.38, Fisher’s exact test). The proportion of case log forms completed during Phase II that incorporated our institution’s assessment platform was 61% (43/70).

**Figure 4:**
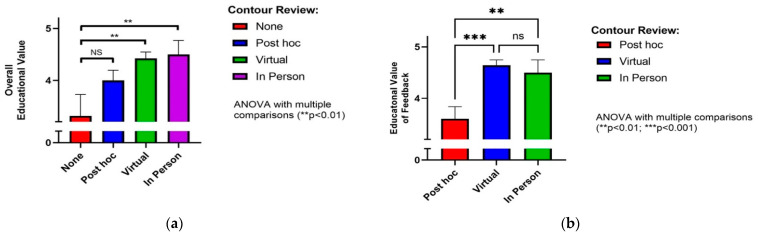
Resident-reported educational values. (**a**) Cases reviewed virtually or in person were statistically ranked higher than those not reviewed. (**b**) Virtual review was ranked significantly more favourable than post hoc review, but similar to in person review. ** *p* < 0.01, *** *p* < 0.001.

We also assessed resident-reported educational value both of the case contouring process overall and the virtual review experience specifically. Residents reported a broad range of learning points or “pearls” across a range of educational themes (Table 1). Resident-reported overall educational value of contouring a case with subsequent virtual contour review was 4.4 ± 0.1 (mean ± standard error, 5-point Likert scale), was no different from in-person review (4.5 ± 0.3, *p* = 0.993), and was significantly higher than for no review (3.1 ± 0.4, *p* = 0.003, ANOVA with multiple comparisons, Figure 4a). The value of immediate feedback during virtual contour review was rated highly among residents (4.6 ± 0.1, mean ± standard error, 5-point Likert scale), no different than ratings for in-person review (4.5 ± 0.2, *p* = 0.803), and significantly higher than feedback received post hoc (e.g., phone or e-mail feedback; 3.6 ± 0.2, *p* < 0.001, ANOVA with multiple comparisons, Figure 4b).

## 4. Discussion

This work describes the development of our resident-led initiative to increase the educational yield of resident contouring during the COVID-19 pandemic. This project was part of an overall response framework to the pandemic by our training program, the full details of which are published separately [6]. After implementing the RECOVR program, we saw significantly increased use of virtual contour review that was highly rated both in overall educational value and in the value of immediate feedback specifically. A key finding was that the perceived educational value of feedback received during virtual contour review was not significantly different from in-person review and was superior to delayed feedback without real-time contour evaluation. To our knowledge, this is the first study to describe a framework for virtual review of contours in a residency program.

Improving consistency of target volume delineation is an active area of research. Atlases and contouring guidelines have been published for various disease sites to reduce variability. Studies examining educational initiatives aimed at teaching anatomy and contouring have also been published [7,8]. Currently, most RO residency programs use an apprenticeship-style model in which residents work one-on-one with staff in clinic and radiotherapy planning. However, there is little published regarding the contour review or feedback process. Previous contouring feedback studies have described using software tools to provide automatic feedback to learners [9,10]. In addition, a recent study described the use of similar videoconferencing software for multidisciplinary peer review of skull base contours during the COVID-19 pandemic [11]. The initiative described using screen sharing functionality to allow review and editing of complex contours with collaboration among RO, otolaryngology, and neuroradiology. However, this was not intended as an educational initiative and did not involve trainees or report on the learning value of the review process.

There has been a recent global shift to competency-based medical education (CBME), in which there is a focus on objectives and abilities, rather than on knowledge or processes [12]. In practice, there is an emphasis on direct observation, frequent assessment, and timely feedback to ensure trainees attain competence. Evidence around CBME and RO education is emerging. The Royal College of Physicians and Surgeons of Canada has published a list of core entrustable professional activities for RO [13], essential tasks that an individual can be trusted to perform once competence has been demonstrated through observed activities. This list acts as a guide for RO education curricula across Canadian training programs, including at our institution. Included in this list are tasks and milestones around radiation treatment planning, such as contouring and treatment plan evaluation. Importantly, trainees are expected to provide rationale behind the contouring process, which is difficult to assess when reviewing contours after the fact rather than in real time. Therefore, we required a process in which real-time review could be performed while observing safety requirements during the pandemic. The RECOVR program ensured that residents continued to develop these core RO competencies. The virtual review process attempted to replicate the in-person experience by allowing direct observation and immediate feedback. Case logs documented educational activity to demonstrate progression. We also embedded a link to our institution’s CBME assessment platform into the case log form to facilitate the assessment process.

Our program was dependent on resident initiative. Trainees were responsible for meeting their weekly targets, and senior residents had the added responsibilities of case assignment. Challenges of program implementation included deployment of the software platform for staff, which required the creation of technical support documents and one-on-one support by experienced users. This program led to transformational change in the contour review process at our institution. Although the case assignment workflow was stopped once apprenticeship rotations were reinstated in July 2020, our program continues to use the virtual contour review process that was introduced through RECOVR.

Strengths of this study include participation of all resident trainees at our institution, representing multiple levels of training. This program used secure software that was available through our institution, avoiding added costs to our program. Limitations included the fact that this initiative was only implemented at a single centre and may not be reproducible in other institutions. For example, larger institutions may face difficulties with software deployment or the coordination of case assignment across multiple sites. Furthermore, we did not explore the impact of this program on learning transfer, behaviours, or performance, and our data were limited to trainees’ perceptions and did not include those of the attendings. Finally, we cannot infer a causal relationship between RECOVR implementation and specific results (e.g., increased case volume or virtual contour review utilization) due to the absence of a control group. Future work on how to optimize feedback during the contour review process would serve to refine the RECOVR program and optimize its educational yield.

## 5. Conclusions

We describe the successful implementation of a remote process for contour review that led to significant increases in contouring and contour review. Our findings provide a data-driven rationale and framework for integrating remote contouring and virtual review into CBME. This approach may provide residents with a novel means of achieving their educational milestones and ultimately attaining the core RO competencies during the pandemic and beyond.

## Figures and Tables

**Table 1:** Example learning pearls grouped by themes.

Theme	N	%	Example Pearls
Contouring	32	42	Review of elective nodal volumes Review of organs at risk for a particular site
Planning considerations	31	40	Considerations in retreatment Dose objectives and constraints Frequency and modality of image guidance Image fusion optimization Rationale of margins for PTV
Clinical	6	8	Criteria for delaying treatment due to the COVID-19 pandemic Review of staging systems Role of chemotherapy
Anatomy	5	6	Review of normal anatomy
Patient set-up	3	4	Indications for wiring Patient positioning

## Data Availability

The data presented in this study are available on request from the corresponding author.
